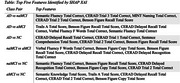# Leveraging Explainable Artificial Intelligence to Identify Key Features required for Differentiating Clinical Neurocognitive Disorder Diagnoses using Toronto Cognitive Assessment

**DOI:** 10.1002/alz70856_105895

**Published:** 2026-01-07

**Authors:** Hamed Azami, Sandra E. Black, Morris Freedman, Stephen C Strother, David F. Tang‐Wai, Carmela Tartaglia, Sanjeev Kumar

**Affiliations:** ^1^ Centre for Addiction and Mental Health, Toronto, ON, Canada; ^2^ University of Toronto, Toronto, ON, Canada; ^3^ Toronto Dementia Research Alliance, Toronto, ON, Canada; ^4^ Hurvitz Brain Sciences Program, Toronto, ON, Canada; ^5^ Division of Neurology, Department of Medicine, University of Toronto, Toronto, ON, Canada; ^6^ Dr. Sandra E. Black Centre for Brain Resilience and Recovery, LC Campbell Cognitive Neurology, Hurvitz Brain Sciences Program, Sunnybrook Research Institute, University of Toronto, Toronto, ON, Canada; ^7^ Sunnybrook Health Sciences Centre, University of Toronto, Toronto, ON, Canada; ^8^ LC Campbell Cognitive Neurology Research Unit, Sunnybrook Research Institute, University of Toronto, Toronto, ON, Canada; ^9^ Sunnybrook Research Institute, University of Toronto, Toronto, ON, Canada; ^10^ Mt. Sinai Hospital, Toronto, ON, Canada; ^11^ Rotman Research Institute, Baycrest Health Sciences, Toronto, ON, Canada; ^12^ Rotman Research Institute Baycrest, University of Toronto, Toronto, ON, Canada; ^13^ Department of Medicine, University of Toronto, Toronto, ON, Canada; ^14^ Krembil Brain Institute, University Health Network (UHN), Toronto, ON, Canada; ^15^ Toronto Western Hospital, Tanz Centre for Research in Neurodegenerative Disease, Toronto, ON, Canada; ^16^ Tanz Centre for Research in Neurodegenerative Diseases, University of Toronto, Toronto, ON, Canada; ^17^ Memory Clinic, Toronto Western Hospital, University Health Network, Toronto, ON, Canada; ^18^ Toronto Dementia Research Alliance (TDRA), Toronto, ON, Canada; ^19^ Department of Psychiatry, University of Toronto, Toronto, ON, Canada

## Abstract

**Background:**

Accurate clinical diagnosis for Alzheimer's disease dementia (AD), amnestic mild cognitive impairment (aMCI), and non‐amnestic MCI (naMCI) is essential for timely management. The diagnosis is made using a range of factors including cognitive testing. Explainable artificial intelligence (XAI)‐based SHAP (SHapley Additive exPlanations) is a machine learning interpretability tool that can provide insights into specific features that drive classification decisions. We used XAI with support vector machines (SVM) to identify key cognitive features of Toronto Cognitive Assessment (TorCA), a user‐friendly cognitive assessment administered by frontline clinicians, for differentiating neurocognitive disorder diagnoses.

**Method:**

We used data from the Toronto Dementia Research Alliance (TDRA) database, comprising of participants with AD, aMCI, naMCI, or normal cognition (NC) seen in memory clinics across Toronto. An SVM model with radial basis function (RBF) kernel was configured with 10‐fold cross‐validation. XAI was integrated using SHAP values to identify the most important critical features contributing to the model predictions. Classification accuracies, defined as the proportion of correct classifications for each pairwise comparison, were calculated using TorCA total scores and specific features from subtests.

**Result:**

We included 695 participants (149 AD, 189 aMCI, 304 naMCI, and 53 NC). Classification accuracy for distinguishing AD vs NC was excellent, whether using all TorCA subtests (0.97±0.03), or the top 5 features (0.96±0.04) (Delayed Recall, Immediate Recall Trials 1 and 2, Sentence Comprehension, Benson Figure Recall), but lower (0.93±0.06) with only TorCA total score. Classification accuracy for MCI or naMCI vs NC was also very good (0.86±0.03 to 0.89±0.04) using the top 5 features.

**Conclusion:**

TorCA combined with XAI can accurately differentiate common clinical neurocognitive disorder phenotypes in ambulatory settings. These findings point to specific cognitive subtests important for diagnosis and may help improve the efficiency of cognitive testing. Future studies should investigate the differentiation of other neurocognitive disorders using these tools and further validate these findings using formal neuropsychological testing.